# Supplementary material for: Vegetation recovery in tidal marshes reveals critical slowing down under increased inundation
Source: Nat Commun. 2017 Jun 9;8:15811. doi: 10.1038/ncomms15811 (PMC5472773; doi:10.1038/ncomms15811)
Supplement: Supplementary Information — Supplementary Figures, Supplementary Tables, Supplementary Notes and Supplementary References [file ncomms15811-s1.pdf]

Supplementary Information

Vegetation recovery in tidal marshes reveals critical slowing down under increased inundation

Jim van Belzen, Johan van de Koppel, Matthew L. Kirwan, Daphne van der Wal, Peter M.J. Herman, Vasilis Dakos, Sonia Kéfi, Marten Scheffer, Glenn R. Guntenspergen & Tjeerd J. Bouma

Supplementary Note 1: Critical slowing down in a simple tidal marsh model

Model description

We developed a simple spatially explicit tidal marsh model adding on ref. [1] to investigate how critical slowing down manifests along the environmental stress gradient elevation due to inundation by seawater. We applied a minimal modelling approach in that we included only those aspects relevant for information of tidal marsh along the inundation gradient, focusing on the coupling of vegetation and sediment elevation. The full model describes changes in vegetation density and elevation along the gradient from land towards the sea by the following partial differential equations:

$$\frac{\partial V}{\partial t} = r\frac{z}{z+a}\left(1-\frac{V}{K}\right)V - m\frac{b}{V+b}\tau(x)V + d_V\Delta V \quad (1)$$

$$\frac{\partial z}{\partial t} = S_{max}\left(1-\frac{z}{z_{max}}\right) - e_{max}\frac{c}{V+c}\tau(x)z + d_z\Delta z \quad (2)$$

Here, *V* and *z* are the vegetation density (g m^{-2}) and elevation (m) respectively and are functions of position *x* and time *t*. The model assumes logistic growth of the tidal marsh vegetation where *r* is the maximum growth rate (d^{-1}) and *K* the maximum standing crop of the vegetation (g m^{-2}). The term *(z/z+a)* is included to account for the positive effect of elevation (higher elevation is lower inundation time) on growth, where *a* is the elevation at which growth is reduced by half. The vegetation is further hampered due to hydrodynamic forcing, such as waves and tidal currents, which increase in importance from the land to the seaward edge of the tidal marsh. We included this as a standardized bottom shear stress $\tau(x)$, which is 0 at the landward and 1 at the seaward edge. The maximum loss rate of the vegetation due to hydrodynamic forcing is *m* (d^{-1}). As the effect of hydrodynamic forcing on the loss of vegetation is density-dependent the term *(b/(V+b))* is included. At high densities, loss of vegetation is reduced due to attenuation and divergence of hydrodynamic energy[1,2] explaining why single seedlings or small tussocks of vegetation on a bare mudflat have little chance of survival[3,4]. The amelioration effect by the vegetation is half maximal at density *b*. Furthermore, we added lateral dispersal of *V* from and to neighboring sites due to colonization via rhizomes, at rate d_v.

The development of the elevation z is determined by sedimentation, erosion and some lateral sediment fluxes. Net deposition is determined by the maximum deposition S_{max} (m d^{-1}), the elevation z (m) and the maximum elevation z_{max} (m) that represents the astronomical high water level the tidal marsh experiences. Simply put, higher elevations are less long inundated by seawater and thus sediments are deposited for shorter period reducing the rate at which the elevation accretes. Sediments can erode due to hydrodynamic forcing along the gradient of bottom shear stress $\tau(x)$ at a maximum E_{max} (d^{-1}). Here, vegetation can reduce erosion e.g. due to attenuation of waves and tidal currents. Erosion is reduced by half at the vegetation density c. Furthermore, there is some diffusive lateral sediment exchange due to e.g. gravity at rate d_z.

Parameter values were obtained from our field experiments, personal observations and literature of similar models[1-4]. The parameter values used for the model analysis were: $r = 2.7*10^{-3}$ day^{-1}, $K = 1000$ g m^{-2}, $a = 1$ m, $m = 2.19*10^{-3}$ day^{-1}, $b = 250$ g m^{-2}, $d_v = 0.012$ m^2 day^{-1}, $S_{max} = 14*10^{-5}$ m day^{-1}, $z_{max} = 4$ m, $e_{max} = 41*10^{-5}$ day^{-1}, $c = 250$ g m^{-2} and $d_z = 0.012$ m^2 day^{-1}.

Estimates for r, K and a were based on our experiments and literature[1,5]. The values for S_{max}, and e_{max} were derived from ref. [1]. All other parameters were based on similar parameters reported in refs. [1-2].

Tidal marsh dynamics were simulated along a 1000 m long cross-shore profile, with 0.25 m discrete sites. The model was initialized by allowing the elevation to reach an equilibrium height without the influence of vegetation (the initial bare tidal flat). This became reference elevation z_0. Next, vegetation is allowed to grow and colonize the bare tidal flat for 100 years before the perturbation experiments are simulated. The new elevation and vegetation density, z_1 and V_1 respectively, serve as a second reference.

Measuring Critical Slowing Down in the model

We focused on how Critical Slowing Down manifests along the environmental stress gradient in the tidal marsh model, comparable to the disturbance-recovery experiments in the field and the time series analysis of the aerial photographs. First we remove 50% of the vegetation biomass of a site to simulate a clipping disturbance. We let the vegetation recover for 100 days (Δt), after which the recovered biomass V_{rec} was obtained.

To simulate the natural disturbance-recovery dynamics due to the erosion of vegetation patches we removed 100% of the vegetation and the elevation was reduced to z_0. We let the vegetation recover for 10 years (Δt). The recovery rates are calculated as (see Supplementary Note 2):

$$\lambda = -log((1-f)/d)/\Delta t \qquad (3)$$

Here f is the relative recovery defined as V_{rec}/V, and d is the magnitude of the vegetation disturbance (here 0.5 for the clipping disturbance and 1 for the erosion disturbance). After a disturbance-recovery experiment is simulated, the procedure is repeated from the initialized vegetation density and elevation (V_1 and z_1) values moving to the next site. The

obtained recovery rates are interpreted using the initial unvegetated elevation z_0 as explanatory variable as this is a proxy for the inundation time.

Simulated results

The model results support that Critical Slowing Down can be found along the elevation gradient (Supplementary Figure 2). For both types of disturbance-recovery experiments we found that the recovery rates decrease from high to low elevations, like we found in our empirical data, and consistent with Critical Slowing Down. Like in our empirical data, the recovery rates of our clipping experiment are a magnitude higher than the recovery from erosion disturbances. These results reveal that tidal marsh resilience differs markedly depending on the type of disturbance, but still in a consistent way along the main stress gradient. Thus, based on the main governing processes modelled in this simple model we can expect Critical Slowing Down to occur along the elevation gradient in real life cases.

Supplementary Note 2: Measuring recovery rates from experimental data

Measuring recovery rates to reliably estimate the resilience in real-world tidal-marsh systems is challenging due to e.g.: 1) difficulty in getting frequent field observations of biomass; 2) seasonal dynamics; and of 3) the high level of heterogeneity and stochasticity in the intertidal system. These above points pose some serious challenges to the usual approached for estimating the recovery rates in perturbed systems. Here, we describe which approaches were used in this study to deal with these issues and obtain reliable estimates on the resilience of vegetation in the field.

Most theoretical and empirical studies follow the nondestructive monitoring approach proposed by ref. [6] for estimating the recovery rate, λ. Here, an exponential model is fitted against the time series of the biomass development that is obtained of the recovery after the disturbance. In the empirical studies that tested resilience indicators so far (e.g. ref. [7, 8]) the state of the system could be relatively easily tracked without disturbing the biomass. For instance, in ref. [7] the researchers used the light attenuated as a proxy for the biomass. By measuring the light attenuation in the mesocosm frequently and at regular intervals the changes in biomass could be tracked; Likewise, in ref. [8] the concentration of cells could be monitored without disturbing the cell numbers. A second method, often used in simulation studies, is to measure the time it takes before the system is recovered to the pre-disturbed state (e.g. used in ref. [9]). The biomass has to be recovered within a certain accuracy around the pre-disturbed value (e.g. within 0.01 or 0.05%) before the disturbance-recovery experiment is stopped.

These approaches are, however, impractical for the assessment of resilience of vegetation in the field. The first methodology (i.e. monitoring of biomass development) requires a series of observations about the vegetation development after disturbance. Tracking biomass (i.e. vegetation) development frequent and at regular intervals is particularly challenging due to the nature of the intertidal environment and the accuracy of the available methods to do so. Due to the tides, accessibility of the field sites shifts from day to day, and from one week to the other, making recurrent field visits logistically difficult. Biomass measures, such as canopy height or coverage are impractical, laborious and can be inaccurate at the small scale of the disturbances due to the high level of variability. Automated observations, e.g. with fixed camera's, still need extensive calibration and might suffer from the harsh hydrodynamic conditions and biofouling. For the second methodology (i.e. monitoring of recovery time) all the above objections remain and are supplemented with the fact that the duration of the experiments is determined by the recovery time, which is impractical. Therefore, an alternative and easy to execute method was required.

To circumvent the above issues, we measured the recovery rate using destructive sampling approach after a fixed time interval consecutive to the disturbance and compared the recovery in the disturbed plot with a proxy for the equilibrium biomass, V_{eq}. This approach is comparable with measuring net primary productivity over a certain time interval (Δt). Like ref. [6], we assume that during the recovery period the development approximates an inverse exponential:

$$V(t) = V_{eq} - V_{eq} D e^{-\lambda t} \qquad (4)$$

Here, $V(t)$ is the biomass at time t, V_{eq} is the equilibrium biomass, and D the disturbance as the amount of biomass removed. To find the recovery rate λ after a fixed time interval Δt we can normalize the function if we define the relative recovery f as $V(\Delta t)/V_{eq}$. In that case the equation writes as:

$$f = 1 - de^{-\lambda \Delta t} \quad (5)$$

in which d is the relative disturbance magnitude, as D/V_{eq}. This leads to the derivation of the recovery rate λ in equation 3.

Finally, we solved the problem that the high level of heterogeneity and stochasticity in the intertidal system poses for the estimation of an equilibrium biomass to which the recovery measurements are related. Due to the high variability of biomass in the field in both the control as well as the experimental plots, pairing them leads to odd results. For instance, this can lead to >100% recovery, which makes it impossible to estimate the recovery rate. However, if we assume that both the control as the experimental plots are sufficiently independent (which is corroborated by the high variability between plots) we can estimate the vegetation distribution parameters (i.e. mean and standard deviation). Therefore, a proxy of equilibrium vegetation V_{eq} was based on the estimate of the maximum biomass of the vegetation per elevation level. This was estimated as the mean biomass of the controls at an inundation level plus 3 times the standard deviation (i.e. the three-sigma-rule).

A full protocol for the disturbance-recovery experiments can be found in ref [10].

Supplementary References

1. van de Koppel, J., van der Wal, D., Bakker, J.P., Herman, P.M.J. Self-organization and vegetation collapse in salt marsh ecosystems. *Am. Nat.* **165**, E1-12 (2005)
2. Bouma, T.J. *et al*. Density-dependent linkage of scale-dependent feedbacks: a flume study on the intertidal macrophyte *Spartina anglica*. *Oikos* **118**, 260-268 (2009)
3. van Wesenbeeck, B.K., van de Koppel, J., Herman, P.M.J., Bouma, T.J. Does scale-dependent feedback explain spatial complexity in salt-marsh ecosystems? *Oikos* **117**, 152-159 (2008)
4. Schwarz, S. *et al*. Abiotic factors governing the establishment and expansion of two salt marsh plants in the Yangtze estuary, China. *Wetlands* **31**, 1011-1021 (2013)
5. Temmerman, S., Bouma, T. J., Govers, G., Lauwaert, D. Flow path of water and sediment in a tidal marsh: Relations with marsh developmental stage and tidal inundation height. *Estuaries* **28**, 338-352 (2007).
6. Van Nes, E. H. & Scheffer, M. Slow recovery from perturbations as a generic indicator of nearby catastrophic shift. *Am. Nat.* **169**, 738-747 (2007).
7. Veraart, A. J. *et al*. Recovery rates reflect distance to a tipping point in a living system. *Nature* **481**, 357-359 (2012).
8. Dai, L., Vorselen, D., Korolev, K. S. & Gore, J. Generic indicators for loss of resilience before a tipping point leading to population collapse. Science **336**, 1175–1177 (2012).
9. Dakos, V., Kéfi, S., Rietkerk, M., van Nes, E. H., & Scheffer, M. Slowing down in spatially patterned ecosystems at the brink of collapse. *Am. Nat*. **177**, E153-E166 (2011).
10. van Belzen, J, van de Koppel, J, Kirwan, M.L, Guntenspergen, G.R, Bouma, T.J. Disturbance-recovery experiments to assess resilience of ecosystems along a stress gradient *Protocol Exchange* doi:10.1038/protex.2017.028 (2017)

Supplementary figures

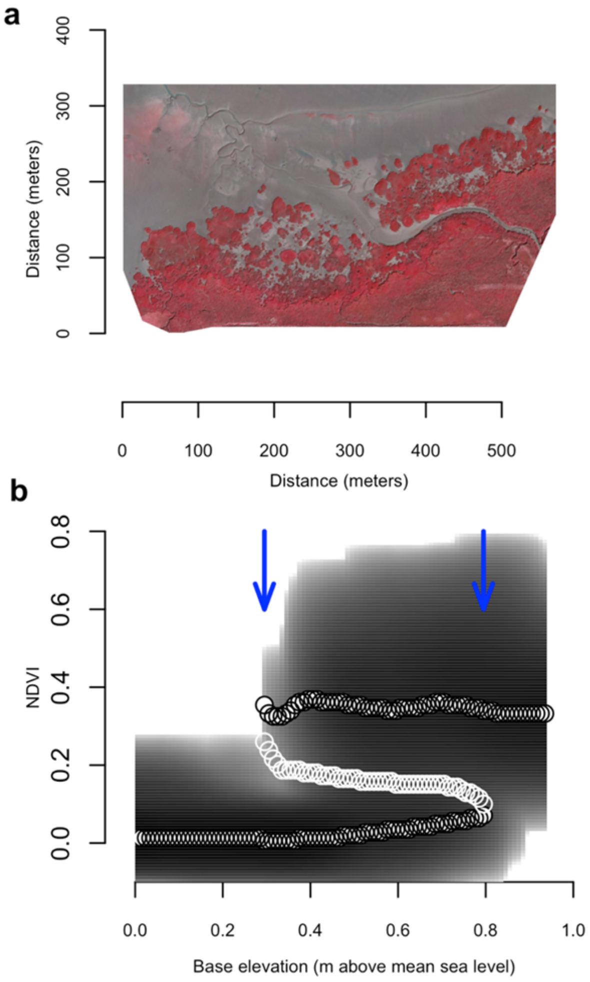

Supplementary Figure 1 | The tidal marsh, and bimodality of vegetation along an environmental stress gradient of seawater inundation.
(a) False color image of tidal marsh vegetation along inundation gradient (red indicates vegetated area) from the sea to land side. **(b)** Reconstruction of the potential (dark gray shading) of the vegetation along the inundation gradient based on the Normalized Difference Vegetation Index (NDVI) suggest the possibility of tipping points in this tidal marsh. The reconstruction indicates a region of bimodality at intermediate inundation times between the high NDVI (biomass) tidal marsh state and a low NDVI (biomass) tidal flat state. White filled and open dots depict these local minima and maxima respectively. Blue arrows depict the critical conditions of marsh vegetation. Panels are based on data from site 2 ‘Paulina’.

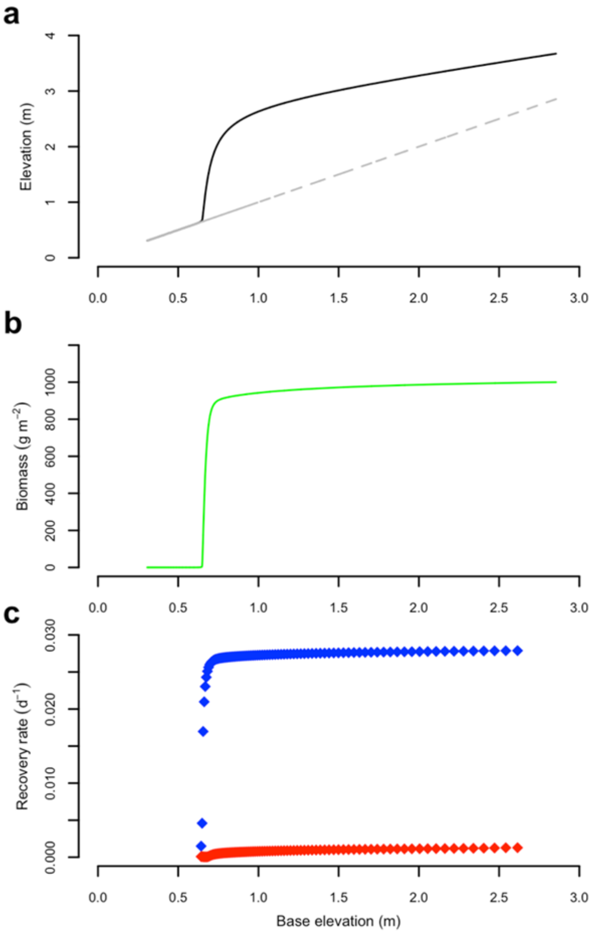

Supplementary Figure 2 | Tidal marsh development and critical slowing down in a simple model.

(a) The equilibrium elevations that establishes along the cross-shore profile in the model. Without vegetation a lower base elevation establishes (dashed grey line) and within the vegetated part of the mudflat the elevation is increased (solid black line). **(b)** Biomass pattern (green line) along the cross-shore profile shows sudden rise in biomass coinciding with the deviating elevation from the base elevation. **(c)** Recovery rates from a simulated clipping disturbance (blue dots) and erosion disturbance (red dots) decrease from high to low elevation indicating critical slowing down.

Supplementary Tables

Supplementary Table 1 | Data available and used of Dutch study sites

year	False Color Composite of aerial images	Bathymetry
1976	1	
1982	1, 2	1*, 2*
1992		1*, 2*
1993	1	1*, 2*
1994		1*, 2*
1996	1, 2	1#, 2#
1997		1#, 2#
1998	1, 2	1#, 2#
2001	1, 2	1, 2
2004	1	1, 2
2008	1, 2	1, 2
2010	1, 2	1, 2
2011	1, 2	?
2012	1, 2	?

1) site 1, Hellegat; 2) site 2, Paulina; *) only data of channels; #) only data of tidal flat adjacent to marsh

Supplementary Table 2 | Correlation between average inundation time and measured indicators of tidal marsh resilience

		**site 1**		**site 2**		**site 3**	
		**Hellegat**		**Paulina**		**Chesapeake: Blackwater**	
	**Parameter**	**r**	**P**	**r**	**P**	**r**	**P**
Remote sensing	Coverage (%)	-0.79	<0.001*	-0.97	<0.001*		
	Recovery rate (d^{-1})	-0.91	<0.001*	-0.77	<0.005*		
	Variance	0.71	<0.001*	0.02	0.96		
	Neighborhood correlation	0.77	<0.001*	0.44	0.13		
Field experiment	Dry mass (g m^{-2})	-0.95	<0.001*	-0.10	<0.001*	-0.33	<0.001*
	Recovery rate (d^{-1})	-0.43	0.02*	-0.53	<0.005*	-0.81	0.008*

*** Pearson's r values are significant**

Supplementary Table 3 | Sensitivity of resilience indicators for the spatial resolution

		**Spatial resolution**							
		**0.25 m**		**1 m**		**5 m**		**10 m**	
**Spatial indicator**	**Site**	**r**	**P**	**r**	**P**	**r**	**P**	**r**	**P**
**Variance**	**#1 Hellegat**	0.71	<0.001*	0.73	<0.001*	0.71	<0.001*	0.69	<0.001*
	**#2 Paulina**	0.02	0.96	0.22	0.47	0.25	0.40	0.24	0.42
**Neighborhood correlation**	**#1 Hellegat**	0.77	<0.001*	0.78	<0.001*	0.76	<0.001*	0.76	<0.001*
	**#2 Paulina**	0.44	0.13	0.49	0.09	0.46	0.11	0.36	0.22

*** Pearson's r values are significant**
